# The rabies distribution pattern on dogs using average nearest neighbor analysis approach in the Karangasem District, Bali, Indonesia, in 2019

**DOI:** 10.14202/vetworld.2021.614-624

**Published:** 2021-03-11

**Authors:** Serli Eka Melyantono, Heru Susetya, Prima Widayani, I Wayan Masa Tenaya, Dinar Hadi Wahyu Hartawan

**Affiliations:** 1 Disease Investigation Centre of Denpasar, Denpasar, Indonesia; 2 Department of Epidemiology and Veterinary Public Health, Faculty of Veterinary Medicine, Gadjah Mada University, Yogyakarta, Indonesia; 3 Department of Geographical Information Science, Faculty of Geography, Gadjah Mada University, Yogyakarta, Indonesia; 4 Disease Investigation Centre of Maros, Maros, Indonesia

**Keywords:** average nearest neighbor, rabies distribution pattern, rabies

## Abstract

**Background and Aim:**

Rabies is a severe progressive encephalitis disease in dogs characterized as a zoonosis. The transmission of rabies between animals in Karangasem District, Bali is still high and continues until today; therefore, rabies in the district still actively circulating. The distribution pattern of rabies, especially in the district, is unknown. This research aimed to describe the spatial distribution of rabies in Karangasem District. The information would help in developing effective control strategies for the disease.

**Materials and Methods:**

An observational study was carried out using 38 positive rabies cases confirmed by the direct fluorescent antibody test diagnosed at the Disease Investigation Centre of Denpasar from September 2018 to September 2019. The Global Positioning System was used to take the geographical coordinates of the places where positive rabies cases had been confirmed in Karangasem District. The ArcGIS version 10.3 (ESRI) was used to determine and analyze the distribution pattern using the average nearest neighbor (ANN) method.

**Results:**

On the basis of the ANN analysis, the rabies distribution pattern in Karangasem District in 2019 was clustered in groups but not significant (Z-score=−1.670309 [<−1.65], p=0.094858 [<0.1]; nearest neighbor ratio=0.858364). The rabies distribution pattern in each subdistrict of Karangasem was dispersed significantly since it had z-score of more than 2.58, p-value less than 0.1 and nearest neighbor ratio of more than 1.

**Conclusion:**

The rabies distribution in Karangasem District had a clustered pattern, although this was not significant. The grouping of rabies in Karangasem District showed a significant dispersed pattern in the subdistricts Abang, Bebandem, and Karangasem. The dispersed pattern of the rabies cases in the subdistricts was caused by unidentified stray dogs that lived in rice fields and other fields and by the whole district’s hilly and mountainous topography. The ANN analysis suggested that for rabies control in Karangasem District, vaccination, elimination, and sterilization of stray dogs should be conducted in densely populated areas.

## Introduction

Rabies is a severe progressive encephalitis disease in dogs or other animals and is characterized as a zoonosis. The infection of this disease is through the bite and/or scratch from infected animals [1]. In Bali, rabies was first introduced in November 2008 by an infected dog owned by a fisherman [2]. Since the first report in 2008, rabies cases have spread to other districts/cities in Bali Province. The prevalence of dogs with rabies in Bali Province in 2019 was 16.94% [3].

Rabies control efforts conducted by the government and Balinese people include massive rabies vaccination programs, stray dog elimination, and educational awareness campaigns [4]. Despite using these strategies to control rabies on the island, the rabies virus has circulated in the province. Spatial analysis on the distribution of rabies in the province will be another effective strategy for controlling the disease.

Rabies case mapping is still limited to a tabular and graphic analysis by aggregating cases at district and subdistrict levels. Identification of individual rabies case’s location can be performed using geographic coordinates with subsequent analyses using the geographical information system (GIS) approach [5]. The GIS is a tool used to analyze data spatially [6]. The spatial analysis would help in giving additional information that cannot be seen clearly on the map. The average nearest neighbor (ANN) method in GIS explains the disease distribution pattern in a location by considering the distance, proximity index, Z-score, and p-value [7]. This method uses an index score to measure the distance between the object’s central spot and other objects’ location. The distribution could be clustered, dispersed, or random.

Rabies cases in Karangasem District in 2019 spread to seven subdistricts, namely, Abang, Karangasem, Manggis, Kubu, Rendang, Bebandem, and Sidemen. The prevalence of rabies is considered high in Karangasem District [3]. Therefore, there is a need to determine the rabies distribution pattern to develop effective control strategies.

This study aimed to determine the spatial distribution of rabies in Karangasem District. Hopefully the information used as a novel control strategy that will subsequently reduce the prevalence of the disease in the district.

## Materials and Methods

### Ethical approval

This study does not require ethical approval as study was based on data only.

### Study type, location and period

The type of research done was observational and held in Karangasem, Bali. The area of Karangasem District is 839.54 km^2^ width and geographically located on 08°33’07”-08°10’00” south latitude and 115°23’22”-115°42’37” east longitude. We used secondary data (positive rabies cases confirmed by FAT) taken from September 2018 to September 2019 (Disease Investigation Centre of Denpasar data), while the study was conducted from October to December 2019.

### Data collection

A total of 38 positive rabies cases were used in this study. All of the cases had been confirmed using direct Fluorescent Antibody Test conducted at The Disease Investigation Centre of Denpasar from September 2018 to September 2019. The geographic coordinates of the exact places where the cases were confirmed were taken using the Global Positioning System (GPS) Garmin Etrex 10.

### Statistical analysis

The GPS data were inputted into MS Excel and converted into shapefile form using the ArcGIS version 10.3 (ESRI) which was used to construct maps and conduct the spatial analysis using the ANN method.

## Results

**Figure-1:**
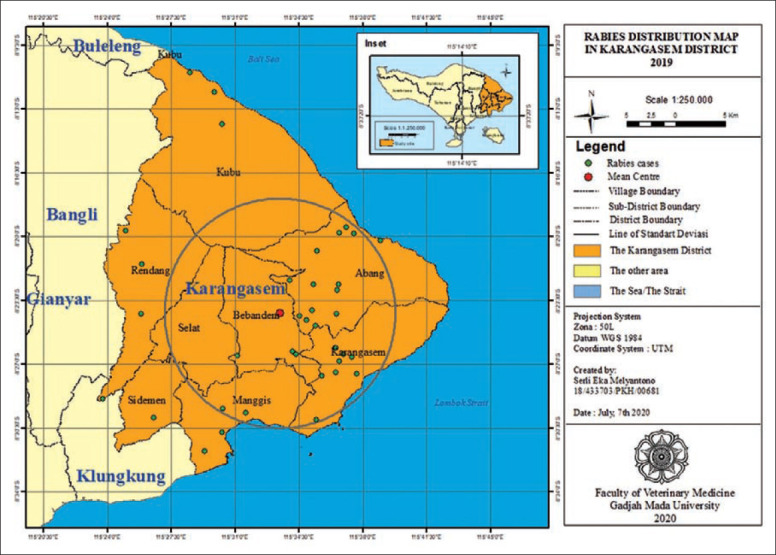
Rabies distribution map in Karangasem District 2019.

**Figure-2:**
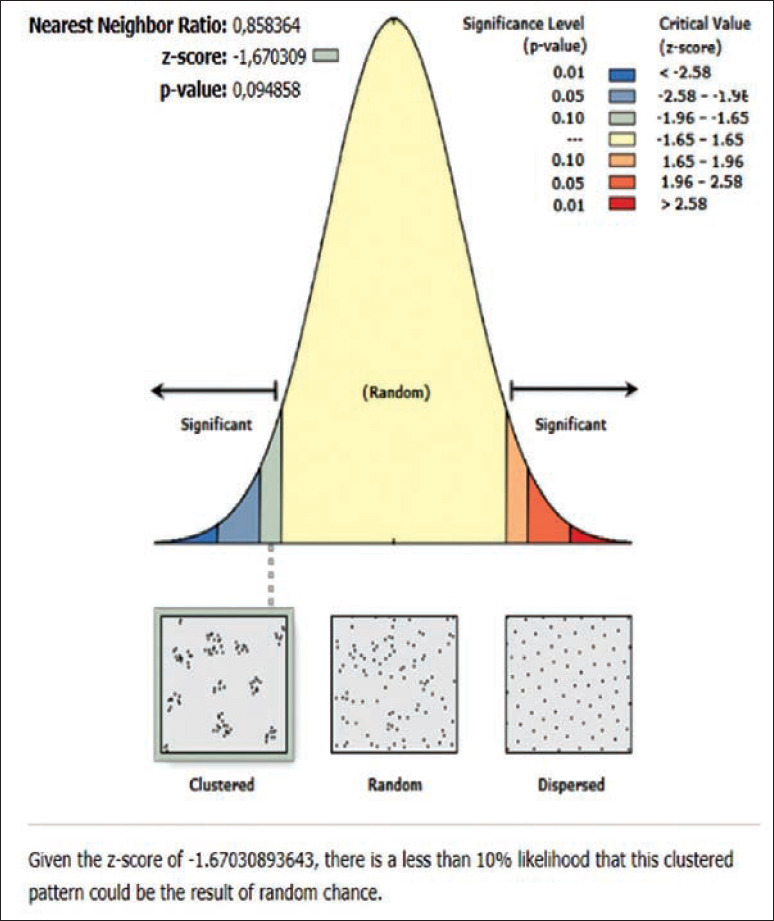
Average nearest neighbor analysis on rabies in Karangasem 2019.

The results of the ANN analysis showed that the rabies distribution pattern in Karangasem District in 2019 was clustered in groups but not significant (Z-score=−1.670309, p=0.094858; nearest neighbor ratio=0.858364; Figures-1 and 2). The result of the ANN analyses in the subdistricts (Abang, Karangasem, Bebandem, and Karangasem) are presented in Table-1. Table-1 shows the subdistricts Abang, Bebandem, Karangasem, Kubu, Manggis and Rendang had Z-score more than 2.58, p-value less than 0.1, and NN ratio of more than 1, thus the distribution pattern of rabies was spreading significantly, while the subdistrict of Sidemen only had one rabies case; therefore the distribution pattern couldn’t be analyzed.

Table-2 shows the total number of rabies cases from 2017 to 2019 in Karangasem District and subdistricts. It was demonstrated that the number of rabies cases increased, and the rural areas had more positive rabies cases in Karangasem District. Rabies was also found in urban areas, including the hinterland areas with a dispersed distribution.

The proportion of areal types in Karangasem District is shown in Table-3.

Figure-1 shows the rabies cases grouping was seen in Abang, Bebandem and Karangasem Districts, while rabies cases in Kubu, Manggis, Rendang and Sidemen Districts were seen to be spreading. The types of areas where rabies cases occur were in the rural, hinterland rural, urban area and hinterland urban types. In Table-3, it can be seen that districts with clustered rabies cases had a high proportion in urban and hinterland urban areas, while the districts with widespread rabies cases had the highest proportion in rural areas. This means that rabies grouping was likely affected by factors in urban and hinterland areas.

**Figure-3:**
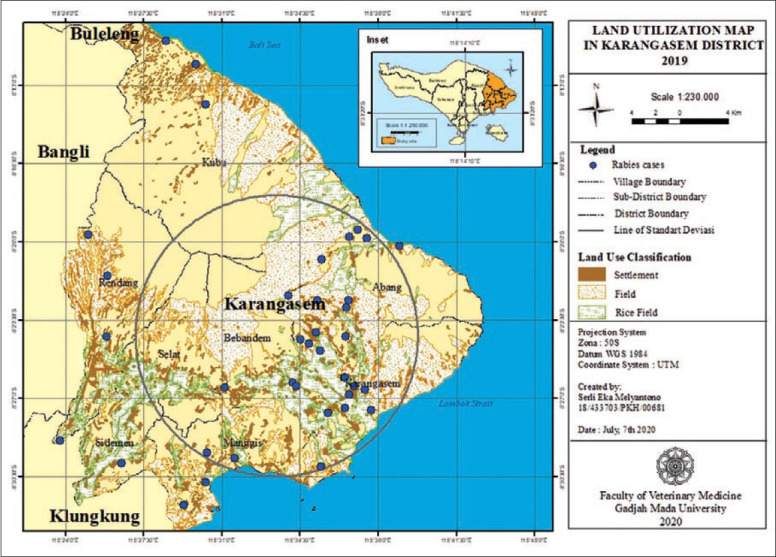
Land utilization in Karangasem District 2019.

Figure-3 shows the land utilization map in Karangasem District in 2019, which was used to categorize rabies distribution. The area is predominantly used for housing, rice fields, and other uses (gardens, forests, and other public facilities like temples, markets, schools, hospitals and government officials).

The rabies distribution map and the results of the ANN analysis in subdistricts Karangasem, Abang, Bebandem, Kubu, Manggis, and Rendang are shown in Figures-4-15. The distribution of the disease shows a dispersal pattern with Z scores of more than 1%.

**Figure-4:**
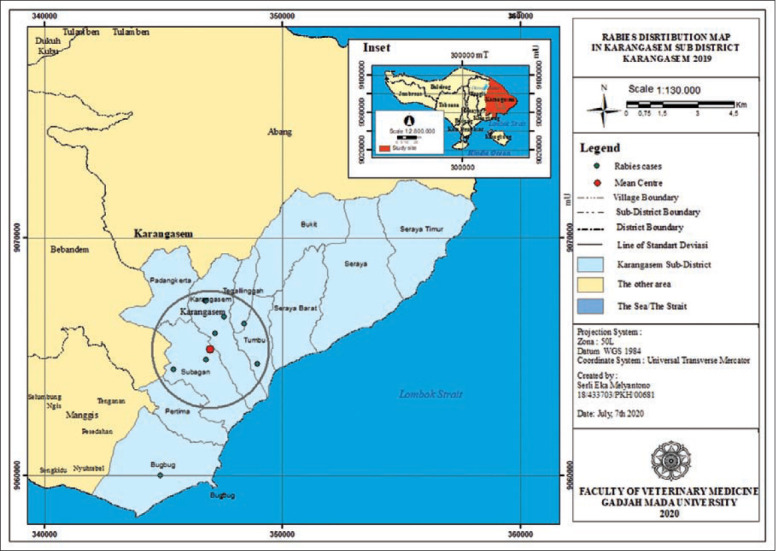
Rabies distribution map in Karangasem subdistrict, Karangasem 2019.

**Figure-5:**
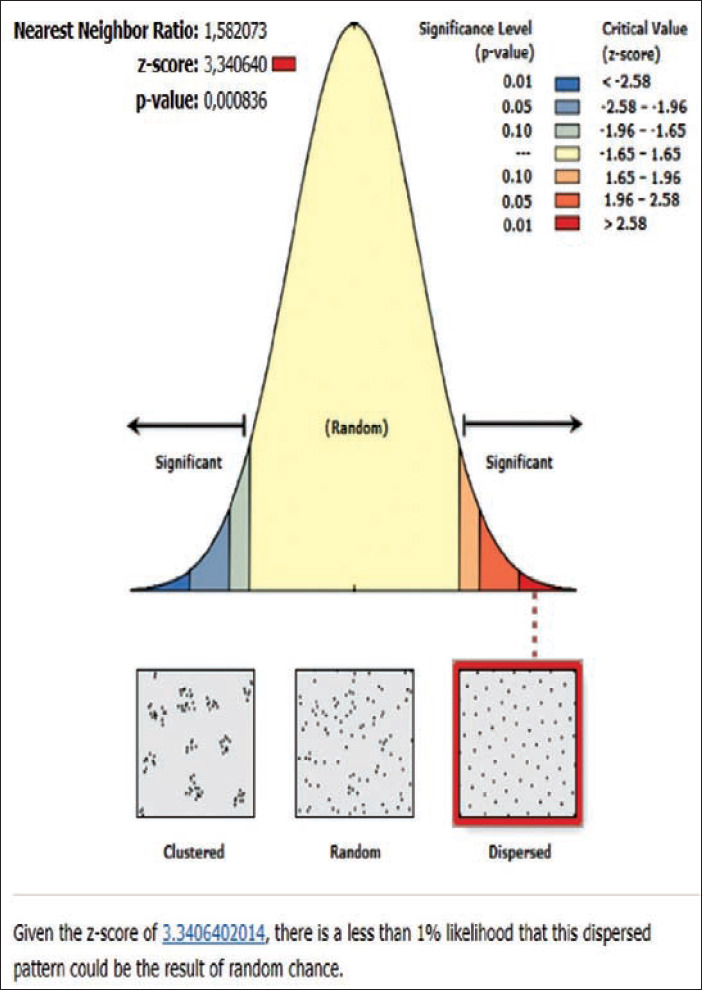
Average nearest neighbor analysis on rabies in Karangasem subdistrict 2019.

**Figure-6:**
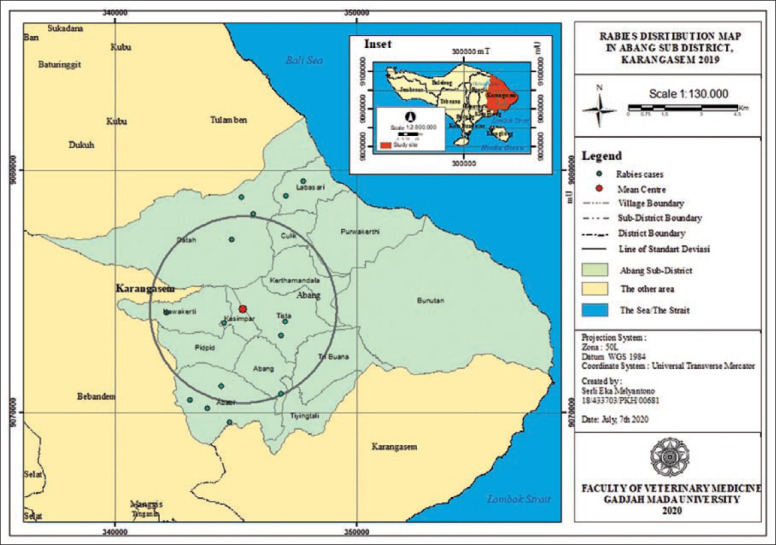
Rabies distribution map in Abang subdistrict, Karangasem 2019.

**Figure-7:**
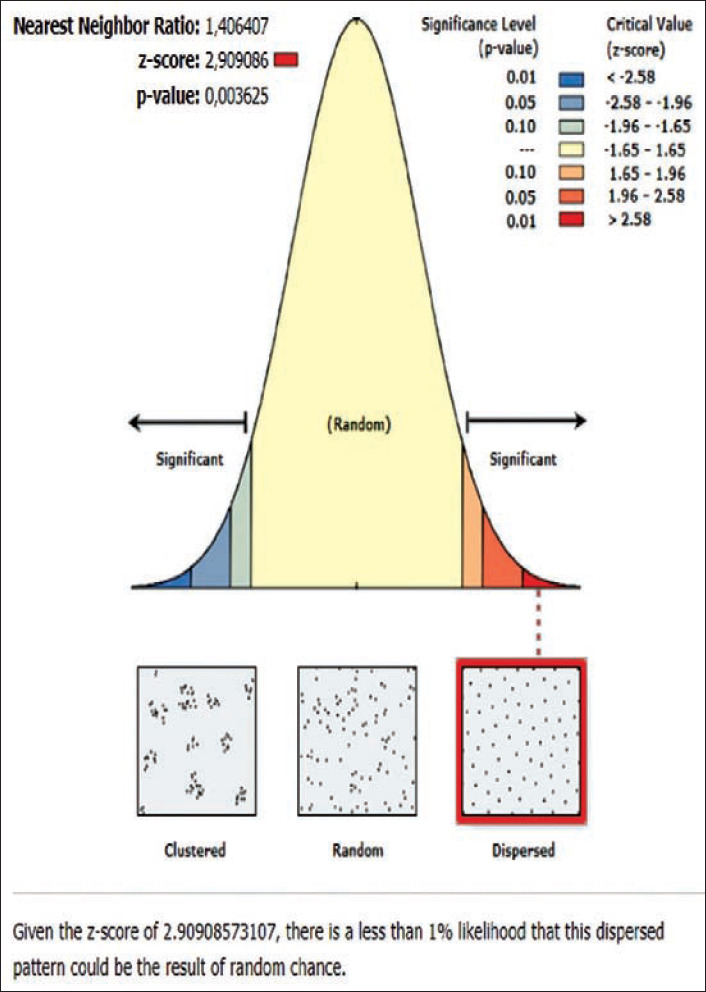
Average nearest neighbor analysis on rabies in Abang subdistrict 2019.

**Figure-8:**
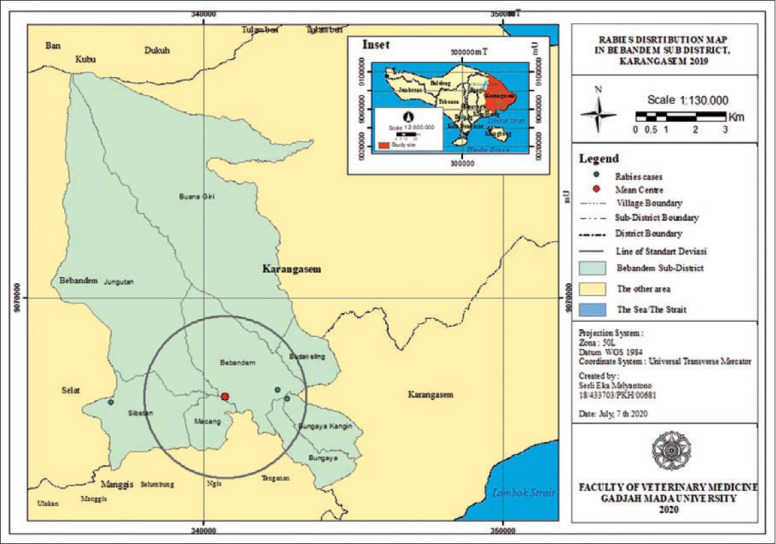
Rabies distribution map in Bebandem subdistrict, Karangasem 2019.

**Figure-9:**
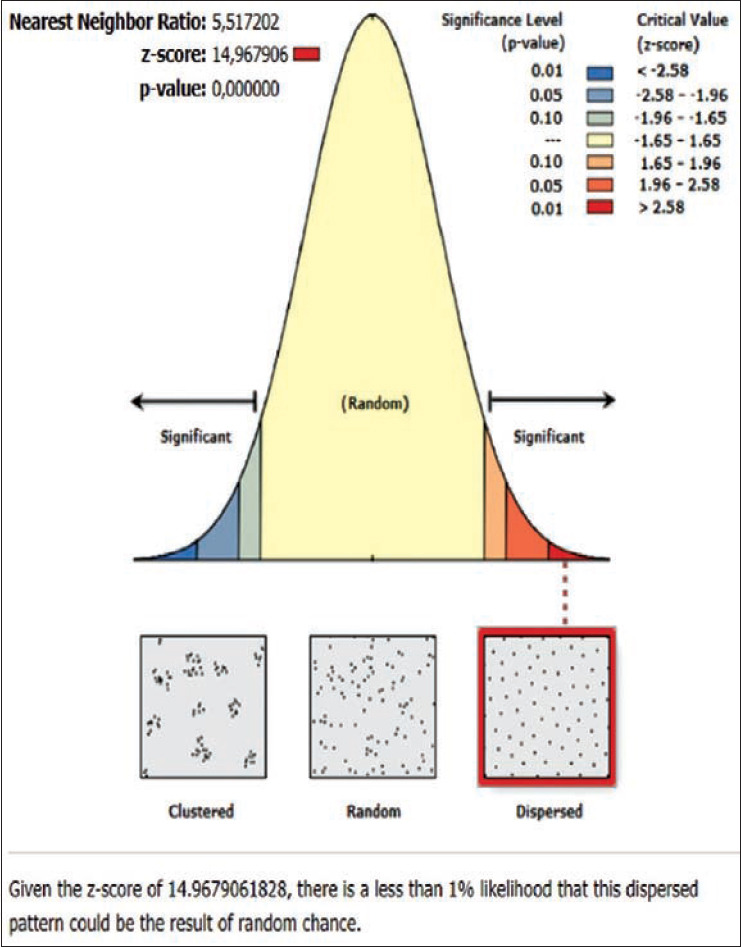
Average nearest neighbor analysis on Rabies in Bebandem subdistrict 2019.

**Figure-10:**
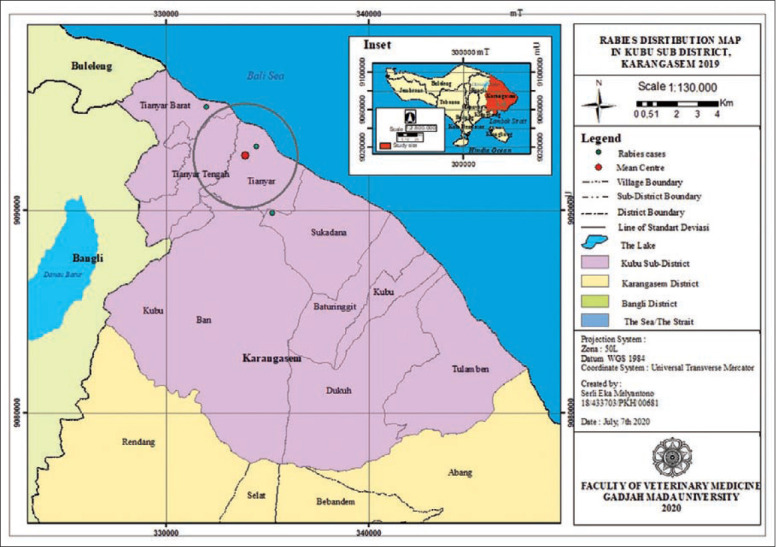
Rabies distribution map in Kubu subdistrict, Karangasem 2019.

**Figure-11:**
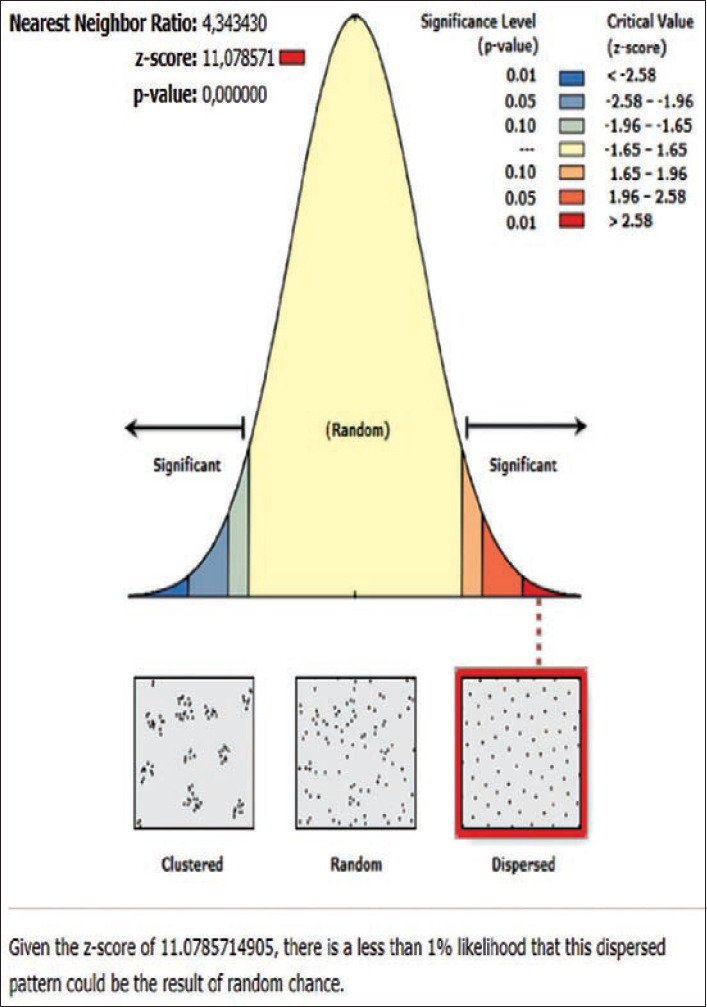
Average nearest neighbor analysis on rabies in Kubu subdistrict 2019.

**Figure-12:**
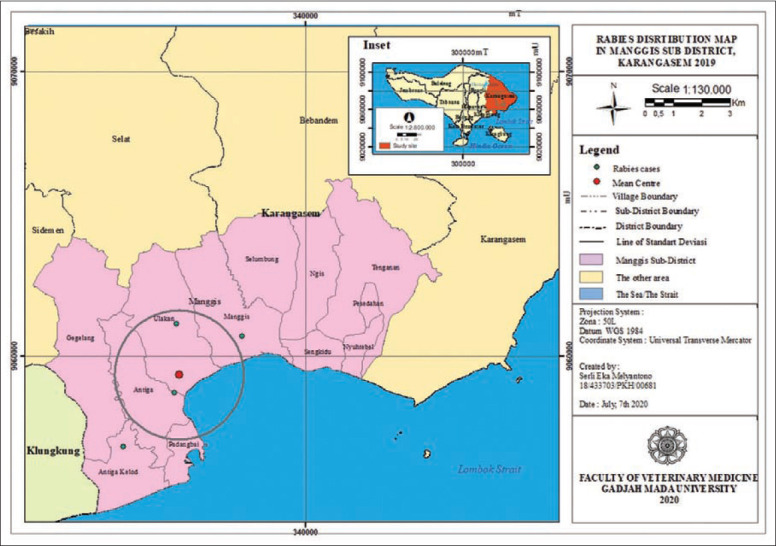
Rabies distribution map in Manggis subdistrict, Karangasem 2019.

**Figure-13:**
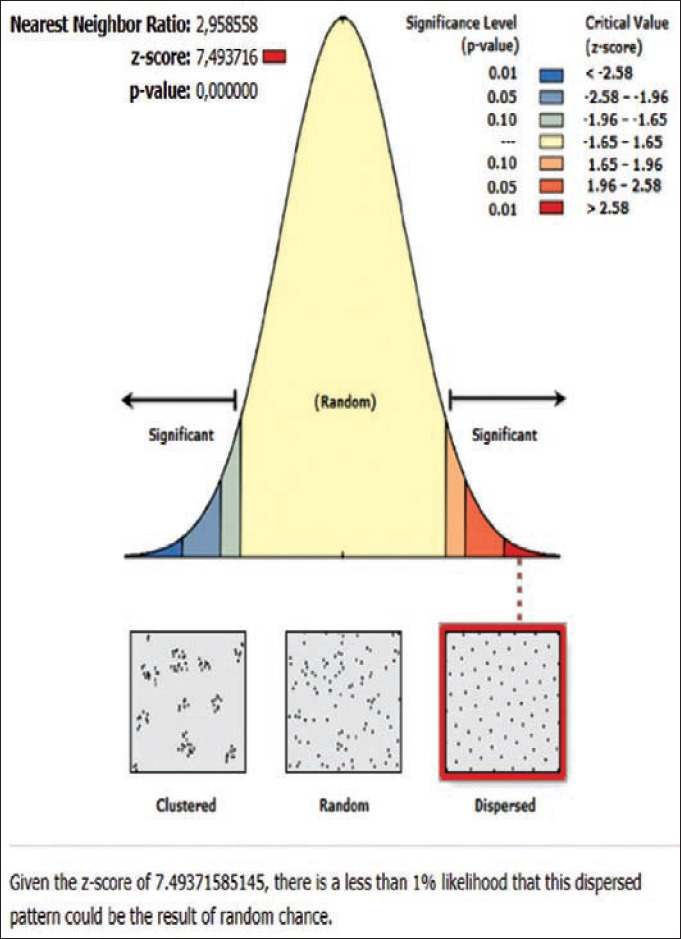
Average nearest neighbor analysis on rabies in Manggis subdistrict 2019.

**Figure-14:**
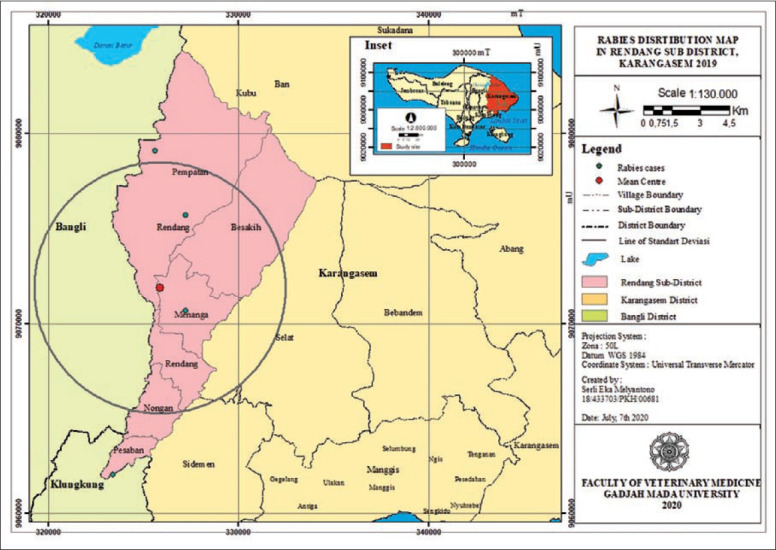
Rabies distribution map in Rendang subdistrict, Karangasem 2019.

**Figure-15:**
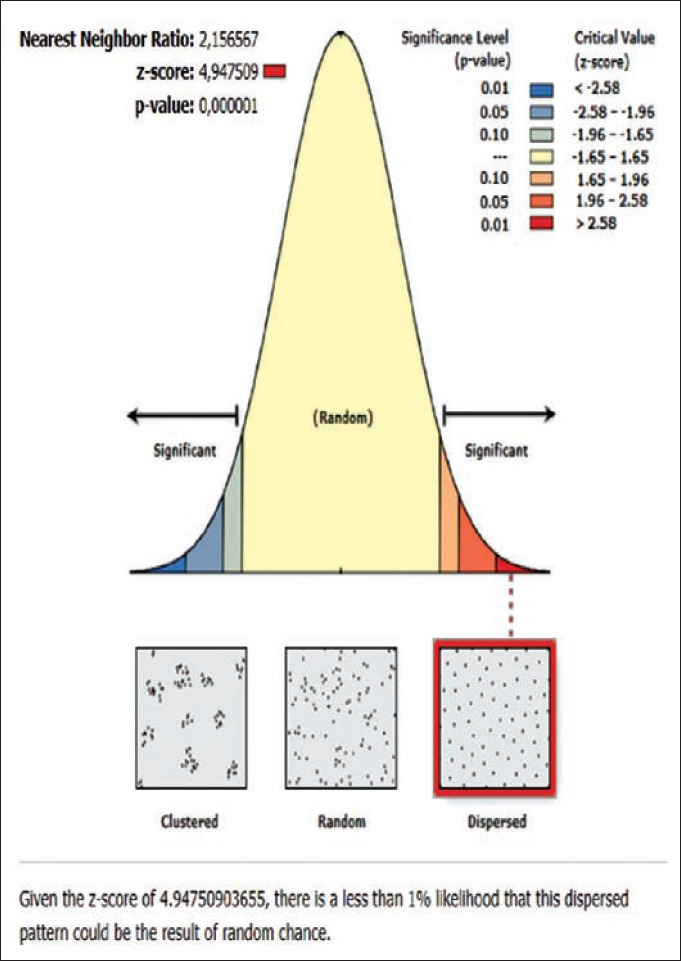
Average nearest neighbor analysis on rabies in Rendang subdistrict 2019.

## Discussion

This study showed that the rabies distribution in the whole of Karangasem District was clustered in groups, and within the subdistricts, a significant dispersed pattern was observed. Of the eight subdistricts in Karangasem, only the Selat subdistrict had no rabies case.

In the subdistricts of Abang, Bebandem, and Karangasem, the clustering observed was probably due to stray dogs’ existence. The prevalence of rabies in stray dogs in those three districts was higher (39%) than in other subdistricts Kubu, Manggis, Rendang, and Sidemen (30%), as reported by Infolab [3]. The positive rabies stray dogs became the source of transmission and spread of disease to other areas. This effect increased rabies cases in the Abang, Bebandem, and Karangasem subdistricts in 2019. After that, most of the transmissions were local in the villages, with more than 2 cases reported within 6 months [8].

In a certain landscape, it was needed to study the existence of suitable habitat host that influences the distribution [9] and the existence [10] of rabies virus. The land utilization map showed that rabies cases were clustered in groups in rice fields and other fields. The stray dog population was high in these areas because there were fewer humans than in settlement areas, and stray dogs would live in a colony, as reported by Mills *et al*. [11]. Dogs tend to search for food in prairie areas rather than in the forest, a protected area [12].

The district of Karangasem is the government center in Karangasem District with a densely populated area [13]. The grouping of rabies cases in Karangasem District was centered in the urban area, including its hinterland. The percentage of dog ownership in Karangasem District was 61.8% [14]. This result means that most people in Karangasem District own dogs, which would increase the risk of rabies transmission. Therefore, controlling the dog population through elimination or sterilization, which theoretically can reduce the dog population, is strongly recommended, as suggested by Taylor *et al*. [15].

Rabies cases in Kubu, Rendang, and Sidemen subdistricts were seen dispersing (outside the circle, as seen in Figure-1); this could be due to transmission or contact from other areas, as they close borders with Bangli and Klungkung District where rabies was also still cycled. Human activity will also influence the rabies distribution pattern. Dog owners who lose their dogs will immediately get new ones, thus facilitating dog movement and increasing the risk of rabies transmission [16].

The number of positive rabies cases was higher in people who released their dogs than those who tethered them. Dogs are released by their owners to guard the house [17]. Releasing dogs will increase the population of largely free-roaming dogs that live close together with their owners [18]. Pepin *et al*. [19] stated that the distance of rabies transmission in skunks is as far as 3.9 km. Releasing dogs as house guards will increase the risk of rabies transmission.

The spread of rabies cases could have come from infected stray dogs that were not vaccinated. A more significant percentage of dogs had not been vaccinated in this study. Dibia *et al*. [20] stated that dogs that have not been vaccinated were sensitive to rabies because they did not have antibodies to fight rabies infection. Those dogs were susceptible rabies infection.

## Conclusion

Rabies distribution in Karangasem District had a clustered pattern, although this was not significant. The clustering of rabies was presumably caused by stray dogs that were infected with rabies. These dogs were commonly found in rice fields and other fields with a small human population. At the subdistrict level, the distribution showed a dispersed pattern. This pattern could be due to the whole district’s topography, which is hilly and mountainous, thus separating the districts. However, because some districts share borders, contact between dogs would occur, leading to disease transmission.

## Authors’ Contributions

SEM desiged and conducted the research. SEM analyzed the result. HS, PW, DHWH, and IWMT corrected and reviewed the manuscript. All authors have read and approved the final manuscript.
